# Clinical significance of serum high sensitive C-reactive protein/albumin ratio in primary prostate biopsy

**DOI:** 10.3389/fonc.2024.1325524

**Published:** 2024-01-31

**Authors:** Xinyang Chen, Yu Li, Gang Li, Xuefeng Zhang, Gansheng Xie, Yuhua Huang, Huming Yin

**Affiliations:** Department of Urology, The First Affiliated Hospital of Soochow University, Suzhou, Jiangsu, China

**Keywords:** high sensitive C-reactive protein-to-albumin ratio, prostate cancer, primary prostate biopsy, Gleason score, BPH

## Abstract

**Objective:**

The purpose of this study was to investigate the clinical significance of serum high sensitive C-reactive protein/albumin ratio in primary prostate biopsy.

**Methods:**

Retrospective analysis was done on the clinical data of 1679 patients who had their first transrectal or perineal prostate biopsy at our situation from 2010 to 2018. Prostate cancer (PCa) and benign prostatic hyperplasia (BPH) were the pathologic diagnoses in 819 and 860 cases, respectively. A comparison was made between the HAR differences between PCa and BPH patients as well as the positive prostate biopsy rate differences between groups with increased and normal HAR. The results of the prostate biopsy were examined using logistic regression, and a model for predicting prostate cancer was created. The receiver characteristic curve (ROC) was used to determine the model’s prediction effectiveness. The clinical models integrated into HAR were evaluated for their potential to increase classification efficacy using net reclassification improvement (NRI) and integrated discrimination improvement (IDI). According to the Gleason score (GS) categorization system, prostate cancer patients were separated into low, middle, and high GS groups. The differences in HAR between the various groups were then compared. The prevalence of high GSPCa and metastatic PCa in normal populations and the prevalence of higher HAR in prostate cancer patients were compared using the chi-square test.

**Result:**

Patients with PCa had a median HAR (upper quartile to lower quartile) of 0.0379 (10^-3^), patients with BPH had a median HAR (0.0137 (10^-3^)), and the difference was statistically significant (p<0.05). Patients with increased HAR and the normal group, respectively, had positive prostate biopsy rates of 52% (435/839)and 46% (384/840), and the difference was statistically significant (p<0.05). Logistic regression analysis showed that HAR (OR=3.391, 95%CI 2.082 ~ 4.977, P < 0.05), PSA density (PSAD) (OR=7.248, 95%CI 5.005 ~ 10.495, P < 0.05) and age (OR=1.076, 95%CI 1.056 ~ 1.096, P < 0.05) was an independent predictor of prostate biopsy results. Two prediction models are built: a clinical model based on age and PSAD, and a prediction model that adds HAR to the clinical model. The two models’ ROC had area under the curves (AUC) of 0.814 (95%CI 0.78-0.83) and 0.815 (95%CI 0.79-0.84), respectively. When compared to a single blood total PSA (tPSA) with an AUC of 0.746 (95%CI 0.718-0.774), they were all superior. Nevertheless, there was no statistically significant difference (p<0.05) between the two models. We assessed the prediction model integrated into HAR’s capacity to increase classification efficiency using NRI and IDI, and we discovered that NRI>0, IDI>0, and the difference was statistically significant (P>0.05).There was a statistically significant difference in HAR between various GS groups for individuals who had prostate cancer as a consequence of biopsy (p<0.05). The incidence of high GS and metastatic patients was statistically significantly greater (p<0.05) in the HAR elevated group (90.1%and 39.3%, respectively) than in the HAR normal group (84.4% and 12.0%).

**Conclusion:**

Prostate biopsy results that were positive were impacted by HAR, an independent factor that increased with the rate of PCa discovery. Patients with elevated HAR had a greater risk of high GS as well as metastatic PCa among those with recently diagnosed prostate cancer through prostate biopsy.

## 1 Introduction

In the US, prostate cancer (PCa) has the highest incidence and second-highest mortality rate among men. In 2019, it’s anticipated that there will be close to 150,000 new instances of PCa in the country, resulting in more than 31,000 deaths (1). The incidence of PCa in China is gradually rising along with the acceptance of screening techniques (2). An increasing number of studies have established a causal link between inflammation and tumors, and pro-inflammatory cytokines, myeloid cells infiltrating the tumor, and immune cells are important players in nearly all stages of the development of inflammation-induced cancer. According to research by Hanahan et al., inflammation can encourage the transformation of cancerous cells. Furthermore, the tumor microenvironment’s inflammatory response plays a crucial role in tumor angiogenesis, metastasis, immune escape, and treatment resistance in addition to aiding in the growth and survival of cancer cells (3–5).

The human liver produces C-reactive protein (CRP), which is one of the frequently used serum biomarkers to assess a patient’s level of inflammation. Research has indicated that patients with malignant tumors suffer various degrees of CRP elevation (6).The process by which CRP rises in cancer patients is associated with the growth and division of tumor cells, which in turn triggers the activation of inflammatory cells and associated components. One possible explanation is that CRP causes angiogenesis to proceed more quickly in cancer patients by elevating their levels of angiogenesis factor and interleukin circulation (7, 8). A high sensitivity CRP detection technique is desperately needed because traditional CRP detection may measure up to 350 mg/L but is unable to detect a minor increase in CRP. High-sensitivity C-reactive protein (hs-CRP) is the term for the process of accurately detecting extremely low concentrations of CRP in the laboratory using ultra-sensitive detection technologies. CRP is a sensitive indication that can be used to discriminate between low- and high-level inflammation (9).The liver also produces albumin (ALB), which is frequently used as a marker of liver function and malnutrition. The primary job of ALB is to keep the plasma colloid osmotic pressure constant in order to maintain the collective blood volume. Reduced serum albumin levels can be caused by infections, burns, liver illness, nephrotic syndrome, and cancers. Low albumin levels, which indicate a lower nutritional condition, are common in cancer patients and are known to interfere with immune systems such humoral and cellular immunity and phagocytosis (10). Albumin is also linked to immunological response and nutritional status in cancer patients. There are even reports that it can diagnose prostate cancer just as well as PSA (11, 12). It has even been claimed that ALB’s diagnostic efficacy for prostate cancer is not weaker than PSA (13). Growing interest has been given to the CAR ratio (C-reactive protein to albumin ratio) as a novel measure of systemic inflammatory response. When assessing a patient’s inflammatory status, it is more useful than ALB and CRP by itself. The prognosis of pancreatic cancer, hepatocellular carcinoma, non-small cell lung cancer, esophageal cancer, gastric cancer, and prostate cancer has been linked to CAR, according to earlier studies (14–19). High sensitive C-reactive protein (hs-CRP) and albumin levels were assessed in 1679 prostate biopsy patients in order to study the association between HAR—high sensitive C-reactive protein to albumin ratio—and prostate cancer to assess HAR’s importance in prostate biopsy.

## 2 Materials and methods

### 2.1 General information

An analysis of 1679 patients who were hospitalized to our hospital between 2010 and 2018 and had their first transrectal or perineal prostate biopsy guided by ultrasonography, confirmed by biopsy pathology, and who satisfied the inclusion and exclusion criteria, was done retrospectively. Inclusion criteria (1): Patients with initial prostate biopsy; (2) No chemoradiotherapy or endocrine therapy was received before puncture;(3) pathologically confirmed Pca or BPH;

Exclusion criteria: (1) Patients with tumors at other sites; (2) Patients with blood system diseases; (3) Patients with infection, inflammatory disease, myocardial infarction and other diseases affecting CRP level within one month before surgery; (4) A history of immune system disease; (5) serious liver and kidney function damage. A total of 1679 patients, aged 28-95 years old with an average age of 68 years old, were enrolled. The median tPSA was 13.85 (8.30~ 26.94) ng/ml, and the median PSAD was 0.34 (0.17~ 0.75) ng/(ml·cm3),the median value of HAR was 0.0351 (0.0147 ~ 0.0996)mg/g.There were 1049 cases of transrectal biopsy, 630 cases of perineal biopsy, 860 cases of BPH, and 819 cases of PCa, of which 602 cases (73.5%) were non-metastatic PCa and 217 cases (26.5%) were metastatic PCa. There were 103 patients (12.6%) with Gleason score ≤6, 292 patients (35.6%) with Gleason score 7 (3 + 4 or 4 + 3), and 424 patients (51.8%) with Gleason score ≥8. According to the median HAR, all cases were divided into normal HAR group (≤0.035mg/g) and elevated HAR group (> 0.035mg/g).This study met the convenient review criteria for retrospective studies of the Medical Ethics Committee of the First Affiliated Hospital of Soochow University, review number: (2021) No. 124.

### 2.2 Methods

After being admitted, the patient was subjected to regular blood sample, tPSA, blood, blood coagulation, and biochemical tests before being biopsyd. tPSA was measured using the chemiluminescence method, and the XT-4000i blood cell analyzer was used to measure leukocyte (WBC), hemoglobin (HB), neutrophil count (N), lymphocyte count (L), monocyte count (M), and platelet count (PLT). The Japanese SysmexCA-7000 hemagglutination equipment was used to quantify fibrinogen (FIB), and the OLYMPUS5400 automatic biochemical instrument was used to measure hs-CRP, ALB, and triglyceride (TG). Total prostate volume (TPV) is equal to 0.52 times the left-right diameter, anterior-posterior diameter, upper and lower diameter, and PSAD is equal to tPSA/TPV.

GE’s Logiq E9 ultra-high-end color Doppler ultrasound diagnostic system and Hitachi’s prior were used to execute ultrasound-guided transrectal or perineal prostate biopsies, respectively, utilizing an 18G automated biopsy needle made in the US by Bard. In both instances, 1-2 needles were placed into suspicious lesions as part of a 12-point (6-point left and right lobes) biopsy system. Both procedures were carried out by urologists. For the purpose of confirming the existence of bone metastases, all patients who had been diagnosed with prostate cancer underwent a bone scan. Additionally, patients whose diagnoses were unclear underwent MR, CT, or PET-CT scans.

PCa was classified into three groups using the GS grading system suggested in the 2014 International Association of Urology Pathology Consensus: low GS group, or GS grade 1, GS=6 points; middle GS group, GS grade 2 and 3, GS=7 points, including GS=3 + 4 points and GS=4 + 3 points; and high GS group, or GS grade 4 and 5, GS=8 points (20). Two doctors with at least three years of experience in prostate pathology rated the GS scores, and if the results were inconclusive, a pathologist with at least five years of experience was consulted.

### 2.3 Statistical treatment

SPSS24.0 was used to conduct the statistical analysis. The measures were indicated as median (upper quartile to lower quartile) and the age distribution was normal, represented as Mean ± SD. The rank sum test was used to compare data with skewness distribution between two or more groups; the c² test was used to compare rates; the t test was used to compare data with normality and homogeneity of variance between two groups; the F test was used to compare the mean of multiple groups with homogeneity of variance; and multivariate Analysis of the correlation between each variable and the prostate biopsy results was done using logistic regression analysis. The combination prediction model of prostate cancer and PSA was compared using the ROC and AUC. The model’s performance was assessed using ROC curve analysis, which yielded AUC. The Delong test was utilized to compare the AUC of several models, with a significance level of P<0.05 being statistically significant. The capacity of various models to increase classification efficiency was assessed using NRI and IDI. Positive improvement is indicated by NRI>0 and IDI>0.

## 3 Results

### 3.1 HAR of PCa patients is higher than that of BPH patients

The median HAR of 819 patients with PCa was 0.0379mg/g (0.0162 ~ 0.1334mg/g), and that of 860 patients with BPH was 0.0311mg/g (0.0137 ~ 0.0830mg/g). The HAR value of patients with PCa was higher than that of patients with BPH. The difference was statistically significant (P < 0.01). There were also significant differences in age, hs-CRP, ALB, hemolymph to monocyte ratio (LMR), tPSA, TPV, PSAD, and HB between PCa and BPH patients (P < 0.01). There were no statistically significant differences in BMI, WBC, PLT, blood neutrophil to lymphocyte ratio (NLR), platelet to lymphocyte ratio (PLR), FIB, and TG (P > 0.05), as shown in **Table 1**.

**Table 1:** Comparison of clinical data between PCa and BPH groups.

Variables	PCa (n=819)	BPH (n=860)	Stats	P -value
Age (years)	71.0 ± 7.8	66.0 ± 8.5	t=-12.60	<0.001
hs-CRP (mg/L)	1.59 (0.69∼4.89)	1.35 (0.60∼3.42)	z=-3.19	0.001
ALB (g/L)	41.80 (38.40∼44.60)	42.80 (39.90∼45.70)	z=-5.59	<0.001
HAR (mg/g)	0.0379 (0.0162∼0.1334)	0.0311 (0.0137∼0.0830)	z=-3.52	<0.001
LMR	3.78 (2.81∼4.90)	4.06 (3.02∼5.37)	z=-3.26	0.001
tPSA (ng/ml)	21.30 (11.49∼48.60)	10.68 (6.93∼16.62)	z=-15.73	<0.001
TPV (ml)	37.67 (25.97∼54.36)	47.93 (32.87∼69.99)	z=-7.40	<0.001
PSAD(ng/ml^2^)((ng/mlpingfen)	0.71 (0.36∼1.40)	0.22 (0.13∼0.40)	z=-17.99	<0.001
HB (g/L)	139 (126∼150)	142 (132∼151)	z=-4.69	<0.001
BMI (kg/m^2^)	23.44 (21.37∼25.39)	23.53 (21.77∼25.35)	z=-1.03	0.304
WBC (10^9^/L)	5.75 (4.90∼6.90)	5.80 (4.86∼7.01)	z=-0.337	0.736
PLT (10^9^/L)	185 (148∼227)	190 (156∼230)	z=-1.533	0.125
NLR	2.36 (1.74∼3.26)	2.26 (1.67∼3.19)	z=-1.560	0.119
PLR	121.37 (93.46∼163.04)	122.36 (91.23∼161.35)	z=-0.244	0.807
FIB (g/L)	2.79 (2.30∼3.40)	2.70 (2.33∼3.30)	z=-1.187	0.235
TG (mmol/L)	1.12 (0.83∼1.59)	1.14 (0.83∼1.63)	z=-0.147	0.883

hs-CRP, highly sensitive C-reactive protein; ALB, albumin; HAR, the ratio of highly sensitive C-reactive protein/albumin; LMR, the ratio of hem lymphocyte to monocyte; tPSA, total prostate-specific antigen; TPV, prostate volume; PSAD, PSA density; HB, hemoglobin; BMI, body mass index; WBC, blood leukocyte; PLT, the platelet count; NLR, the ratio of neutrophils to lymphocytes; PLR, the ratio of platelets to lymphocytes; FIB, fibrinogen; TG, triglyceride.

### 3.2 Influence of elevated HAR on the positive rate of prostate biopsy.

There were 839 patients with elevated HAR (> 0.0351mg/g), including 435 patients with PCa, with a positive rate of 52%. There were 840 patients with normal HAR (≤0.0351mg/g), including 384 patients with PCa, with a biopsy positive rate of 46%, the difference between the two was statistically significant (χ²=6.319, P < 0.05), as shown in **Table 2**.

**Table 2:** Comparison of clinical data between the elevated HAR group and the normal group.

Variables	HAR>0.0351mg/g (n=839)	HAR ≤ 0.0351mg/g (n=840)	stats	P
Age (years)	69.7 ± 8.7	67.2 ± 8.2	t=6.14	<0.001
tPSA (ng/ml)	16.09 (9.20∼33.24)	12.36 (7.64∼21.67)	z=-5.68	<0.001
TPV (ml)	44.14 (29.63∼65.73)	49.21 (29.48∼63.18)	z=-1.23	0.220
PSAD (ng/ml2)	0.37 (0.18∼0.88)	0.31 (0.16∼0.66)	z=-2.47	0.014
Biopsy positive rate	52% (435/839)	46% (384/840)	χ²=6.32	<0.05

tPSA, total PSA; TPV, prostate volume; PSAD, PSA density.

### 3.3 Logistic regression analysis of HAR and prostate biopsy results and the combined prediction model of prostate cancer

**Figure 1:**
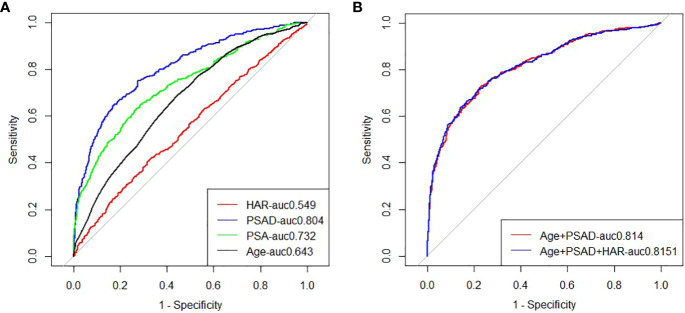
**(A)** ROC analysis of clinical risk factors predicting prostate biopsy for prostate cancer; **(B)** ROC analysis of clinical risk factors alone or in combination with HAR to predict prostate cancer from primary prostate biopsy.

Logistic regression study revealed that the good outcomes of prostate puncture were independently influenced by HAR (OR=3.391, 95%CI 2.082-4.977, P < 0.05), PSAD (OR=7.248, 95%CI 5.005-10.495, P < 0.05), and age (OR=1.076, 95%CI 1.056 ~ 1.096, P < 0.05).,as shown in **Table 3**.The area under the curve for PCA was estimated by clinical models based on age and PSAD to be 0.814 (95%CI 0.78-0.83). The AUC of a comprehensive model that took age, PSAD, and HAR into account was 0.815 (95%CI 0.79-0.84). They were all better than a single blood total PSA (tPSA) with an AUC of 0.746 (95%CI 0.718-0.774),as shown in **Figure 1**.The results of the Delong test revealed that there was no statistically significant difference (P>0.05) between the two models. The potential of the comprehensive model to enhance the classification effect was further assessed using NRI and IDI. Positive improvement capacity was observed (NRI>0, IDI>0) in comparison to the single clinical model, and the difference was statistically significant (P<0.05),as shown in **Table 4**.

**Table 3:** Logistic regression analysis of each variable and the results.

Variables	Regression	Wald	P	OR	95%CI
Age (years)	0.073	61.461	0.000	1.076	1.056∼1.096
HAR (mg/g)	1.221	4.081	0.043	3.391	2.082∼4.977
PSA (ng/ml^2^)	1.981	109.959	0.000	7.248	5.005∼10.495
tPSA (ng/ml)	-0.001	0.329	0.566	0.999	0.994∼1.003
LMR	0.019	0.354	0.552	1.020	0.956∼1.087
HB (g/L)	0.005	1.087	0.297	1.005	0.996∼1.013

HAR, the ratio of highly sensitive C-reactive protein/albumin; PSAD, the PSA density; tPSA, the total PSA; LMR, the ratio of hem lymphocyte to monocyte; HB, hemoglobin.

**Table 4:** Analysis of the increased predictive value of HAR in the diagnosis of Pca by primary prostate biopsy.

Variables	Clinical model	comprehensive model	P-value
NRI	–	0.0171(-0.0142∼0.0484)	0.285
IDI	–	0.0052(0.0013∼0.0091)	0.009

### 3.4 Differences in variables between different GS groups

Except for Age and LMR, there were statistically significant differences in all indexes among different GS groups (all P < 0.05), as shown in **Table 5**.

**Table 5:** Differences of clinical indicators among different GS groups.

Variables	Gleason groups		stats	P-value
	Low-medium GS group (n=395)	High GS group (n=424)		
Age (year)	70.56.97 ± 7.61	71.46 ± 8.00	t=-1.639	0.101
HB (g/L)	142 (130∼151)	136 (122∼147)	z=-5.041	0.000
hs-CRP (mg/L)	1.27 (0.60∼3.34)	2.25 (0.82∼7.70)	z=-4.953	0.000
ALB (g/L)	42.3 (38.7∼45.0)	41.5 (38.1∼43.8)	z=-3.177	0.001
HAR (mg/g)	0.0293 (0.0139∼0.0847)	0.0504 (0.0193∼0.1937)	z=-5.006	0.000
tPSA (ng/ml)	15.59 (6.93.18∼30.71)	32.59 (17.54∼64.31)	z=-9.379	0.000
PSAD (ng/ml^2^)	0.54 (0.26∼1.14)	0.92 (0.50∼1.81)	z=-5.869	0.000
TPV (ml)	35.22 (23.67∼53.87)	38.33 (28.78∼54.98)	z=-2.208	0.027
LMR	3.76 (2.81∼4.85)	3.84 (2.81∼5.03)	z=-0.366	0.714

HB, hemoglobin; hs-CRP, highly sensitive C-reactive protein; ALB, albumin; HAR, the ratio of highly sensitive C-reactive protein to albumin; tPSA, total PSA; PSAD, PSA density; TPV, prostate volume; LMR, the ratio of hem lymphocyte to monocyte.

### 3.5 Differences in the proportion of PCa with high GS and metastatic PCa between the elevated HAR group and the normal group

Among the patients with prostate cancer whose initial biopsy results were obtained, the proportion of PCa with high GS in the HAR elevated group was 91.3%, the proportion of PCa with metastatic disease was 53.8%, the proportion of PCa with high GS in the HAR normal group was 85.4%, and the proportion of PCa with metastatic disease was 11.8%, both of which were higher in the HAR elevated group than in the HAR normal group (P < 0.05), as shown in **Table 6**.

**Table 6:** Comparison of the proportion of high GS and metastatic PCa in patients.

Variables	HAR>0.0351 mg/g (n=435)	HAR ≤ 0.0351 mg/g (n=384)	Stats	P-value
High GS for PCa ratio	90.1% (392/435)	84.4% (324/384)	χ²=6.112	0.013
Metastatic PCa ratio	39.3% (171/435)	12.0% (46/384)	χ²=78.228	<0.001

## 4 Discussion

Chronic inflammation and malignancies are intimately connected, and CRP is one of the most researched inflammatory indicators (21). In past research, we discovered that patients with high hs-CRP were more likely to have a prostate biopsy result that was positive and to have bone metastases (22, 23). According to a recent prospective study called PROCA-life, PCa risk and prognosis were worse in male patients with elevated hs-CRP (24). In this study, hs-CRP was also significantly greater in the PCa group than in the BPH group, and it was also significantly higher in the PCa group with high GS than in the PCa group with low and middle GS. Currently, it is thought that tumors and inflammation interact and have an impact on one another: Numerous factors that cause chronic inflammation, including amyloid (which causes physical damage), high-fat diet consumption, obesity, chemical damage, and intestinal and urinary tract microbes all contribute to the occurrence and development of prostate cancer in various ways (25). Most of these inflammatory reactions involve CRP. Inflammatory cytokines like interleukin-1 and interleukin-6, which act on the liver to produce hs-CRP, can be produced by inflammatory cells in the tumor microenvironment (26). According to the well-known Swedish AMORIS trial, ALB levels were found to be favorably associated with GS 4 + 3 prostate cancer and negatively associated with high-risk or metastatic prostate cancer 14 years before diagnosis (27). ALB in this study was shown to be significantly lower in the PCa group than the BPH group and to be significantly lower in the PCa group with high GS than the PCa group with low and intermediate GS. Most studies indicate that albumin is a key indication of the prognosis of tumor patients and that it is the primary reflection of the nutritional condition of tumor patients. The prognosis of patients is often poor when the nutritional status is poor at the advanced stage of the malignancy. Although nutritional status is typically unaffected before or in the early stage of tumor incidence, certain studies have revealed that albumin declines before or in the early stage of tumor occurrence (27, 28). We hypothesize that albumin contributes to the development of tumors or to its early stages. Currently, there are primarily two hypotheses that potentially account for this association between albumin and tumors: First, inflammatory mediators make capillaries more permeable, which results in intravascular albumin leakage into the tissue space (29). Second, decreased albumin production may be caused by interleukin-6, tumor necrosis factor, and acute phase reactants (30). In their initial investigation into the connection between CAR and prognosis in castration-resistant prostate cancer (CRPC), Kazumasa Komura et al. discovered that individuals with high CAR had shorter tumor-specific survival for CRPC patients treated with abiraterone or enzalutamide. In terms of predicting both overall and tumor-specific survival, CAR is an independent predictor.

For the first time, the relationship between HAR prior to the initial prostate biopsy and the biopsy results was examined in this study. It was discovered that HAR prior to the biopsy was highly correlated with the prostate biopsy positive rate, Gleason score, and distant metastasis of prostate cancer, indicating that HAR may be linked to the aggressiveness and malignancy of prostate cancer. Our findings confirm the two aforementioned theories about albumin and tumor, and they also explain why patients with high HAR in other studies had a worse prognosis. Selection bias could have resulted from this study’s single-center retrospective design and lack of external cohort validation. Serum HAR levels can be influenced by a wide range of circumstances. However, because these confounding factors could not have been controlled for in this investigation, the results may be biased. Second, in order to increase the prediction efficacy of the model, we neglected to incorporate prostate MRI and free/total PSA in our analysis because of the lengthy duration and dearth of clinical data. The lack of follow-up among the prostate cancer patients made it impossible to assess the correlation between HAR and the disease’s prognostic value or its association with survival, progression, or recurrence. Therefore, in order to confirm the findings of this investigation and provide greater insight into the diagnostic and prognostic utility of HAR for patients with prostate cancer, larger sample sizes, multi-center studies, and prospective designs are required in the future.

Serum PSA detection is cost-effective, practical, and less invasive. It has a high sensitivity but a low specificity, which leads to more unnecessary biopsies, particularly as the number of instances of clinically meaningless PCa rises year after year. Although it may be connected to the aberrant rise in PSA in the majority of the cases in this study, PSA is not a standalone factor in this study that influences the outcomes of prostate biopsy. Prostate inflammation, prostate volume, and transrectal digital examination of the prostate are additional factors contributing to the rise in cases of prostate cancer. Some investigations even reported that there was no statistical difference in PSA between prostate cancer and prostate hyperplasia in men with prostate biopsy (31). Occasionally, PSA struggles to discriminate these disorders efficiently. Although the development of multi-parameter magnetic resonance and targeted biopsy technology in recent years has increased the detection rate of clinically significant PCa (32, 33), these two technologies cannot currently be used in the majority of primary hospitals due to financial constraints and technical limitations. Therefore, tPSA, PSAD, and age should also be taken into account while evaluating the outcomes of prostate biopsy in clinical work. HAR is another option.

## Data availability statement

The raw data supporting the conclusions of this article will be made available by the authors, without undue reservation.

## Ethics statement

The studies involving humans were approved by the Medical Ethics Committee of the First Affiliated Hospital of Soochow University. The studies were conducted in accordance with the local legislation and institutional requirements. Written informed consent for participation was not required from the participants or the participants’ legal guardians/next of kin because the risk of the study is minimal and the consent of the subject cannot be obtained. This study met the convenient review criteria for retrospective studies of the Medical Ethics Committee of the First Affiliated Hospital of Soochow University, review number: (2021) No. 124.

## Author contributions

HY conceived the idea. HY designed the study. XC, YL, GX, GL and XZ collected and analyzed the data. XC,YL and YH provided methodology. XC and YL drafted the manuscript. HY and XC reviewed and corrected the manuscript. All authors contributed to the article and approved the submitted version.
